# Varicella-associated disseminated intravascular coagulation secondary to Henoch-Schönlein purpura with renal and gastrointestinal system involvement in a child: A case report

**DOI:** 10.1097/MD.0000000000036203

**Published:** 2023-11-17

**Authors:** Jing Jiang, Kai Liao, Hui Guo, Xiu-Ying Chen

**Affiliations:** a Department of Pediatrics, West China Second University Hospital, Sichuan University, Chengdu, Sichuan Province, China; b Key Laboratory of Birth Defects and Related Diseases of Women and Children (Sichuan University), Ministry of Education, Chengdu, Sichuan Province, China; c Department of Radiology, West China Hospital, Sichuan University, Chengdu, Sichuan Province, China.

**Keywords:** case report, disseminated intravascular coagulation, gastrointestinal involvement, Henoch-Schönlein purpura, varicella zoster virus

## Abstract

**Rationale:**

Immunocompromised patients who developed varicella-zoster virus (VZV)-associated disseminated intravascular coagulation (DIC) previously included recipients of bone marrow, hematopoietic stem cell, or organ transplantations, patients with primary nephropathy receiving corticosteroid therapy, cancer patients receiving chemotherapy, and patients with human immune deficiency virus infection. The case reported here is novel because, to our knowledge, there has been no report of VZV-associated DIC after the onset of Henoch-Schönlein purpura (HSP).

**Purpose:**

To report the successful treatment of a novel pediatric case with VZV-associated DIC secondary to HSP.

**Diagnosis and intervention:**

An 8-year-old girl developed VZV-associated DIC 24 days after diagnosis of HSP with renal and gastrointestinal involvement. She was treated with methylprednisolone at a local hospital for 19 days, and suddenly developed fever starting from day 4 in our hospital. Her fever persisted with vesicular skin rashes on her back, strong abdominal and lower back pain, epistaxis, hematochezia, erosion and bleeding on her lips, in her mouth and at puncture sites on day 5. She was diagnosed with DIC with the laboratory evidence of dramatically decreased platelet count and fibrinogen, prolonged activated partial thromboplastin time and prothrombin time, and increased fibrin degradation products including d-dimers. She also developed multiple organ dysfunction syndrome. On day 7, the patient VZV nucleic acid result turned out to be positive. Methylprednisolone treatment was discontinued, and she was given a multi-modality therapy including medications of acyclovir and antibiotics, intravenous gamma-immunoglobulin, various blood product transfusions, continuous renal replacement therapy, plasma exchange, and administration of liver and gastrointestinal system protection drugs.

**Outcomes:**

The patient multi-organ function damage gradually recovered. After VZV control, the patient was treated with oral methylprednisolone again for HSP with nephritis. Urine analysis was normal 1 year later, and oral hormone was discontinued. No complication or relapse occurred during 2 years of follow-up.

**Significance:**

This case report, for the first time, adds HSP treated with corticosteroids to the spectrum of clinical conditions that progressed to life-threatening secondary varicella-associated DIC. Early identification of varicella infection and DIC, combined with timely antiviral, immunoglobulin transfusion, plasma exchange, and other combined therapies are essential for saving patients’ lives.

## 1. Introduction

Varicella (chicken-pox) is an acute infectious disease caused by varicella-zoster virus (VZV) infection. It usually occurs in children in winter and spring. Although varicella is mostly a mild and self-limiting disease, it can result in severe complications, which were a major impetus towards varicella vaccine development. Severe varicella caused many deaths in individuals with impaired immunity, even after the development of antiviral therapy.^[1,2]^ Data from developed countries showed that ~5 out of 1000 people with varicella were hospitalized and 2 to 3 per 100,000 patients died.^[2]^ Common complications of varicella include bacterial sepsis, pneumonia, encephalitis and hemorrhagic manifestations.^[1,2]^ Immunocompromised patients or individuals who need to use corticosteroids or immunosuppressants may be more vulnerable to VZV infection, which may cause systemic and disseminated toxemia, leading to hyper-inflammatory syndrome, multiple organ damage, and death.^[3–17]^ One of the severe varicella complications is disseminated intravascular coagulation (DIC), which has a high risk of fatal outcomes.^[3–5]^ However, childhood varicella-associated DIC secondary to Henoch-Schönlein purpura (HSP) has never been reported. Here we report, for the first time, the diagnosis and treatment of a rare childhood case of secondary varicella-associated DIC that developed 24 days after the initial diagnosis of HSP with renal and gastrointestinal system (GIS) involvement. After prompt aggressive treatment, the child gradually recovered. Clinicians should be cautious with secondary life-threatening varicella in immunocompromised populations including HSP patients receiving corticosteroid treatment. Early diagnosis and intensive multi-modality treatment are crucial for saving the patients’ lives.

## 2. Case presentation

The patient was an 8-year-old girl transferred to our hospital (West China Second University Hospital of Sichuan University) from a local hospital. Nineteen days prior to the transfer, she was admitted to the hospital for palpable purpuric rash, vomiting, and abdominal pain. Routine blood test and coagulation function test results were normal; urinalysis indicated hematuria 3+, proteinuria 2+; and abdominal color Doppler revealed intussusception. The patient had no history of contact with people with known chicken-pox and was healthy before the onset of the disease. There was no family history of special diseases. Viral serology and biopsy of the skin and kidney were not performed at that hospital. She was diagnosed with HSP with nephritis and intussusception. After treatment with methylprednisolone, cimetidine, vitamin C, loratadine, air enema and fluid infusion, the clinical conditions of abdominal pain and vomiting improved, and the skin rash appeared to be slightly subsided. For further treatment of her disease, she was transferred to Nephrology Department of our hospital with the presentation of bilaterally swollen ankle joints, periumbilical tenderness, palpable purpuric rash on her face, auricle, eyelids, the backs of both hands, and obsolete palpable rash on bilateral lower limbs, without color-fading when pressed. Physical examination on admission showed a body temperature of 36.7°C, respiratory rate 20/minute, heart rate 85 beats/minute, and blood pressure 98/60 mm Hg. Examination of heart and lung was unremarkable. The representative laboratory results are listed in Table 1. Urinalysis showed hematuria 3+, proteinuria 4+, and pathological casts. Antinuclear antibodies, anti-dsDNA, anti-neutrophil cytoplasmic antibodies, anti-cardiolipin antibodies, anti-protein C and anti-protein S antibodies, and tumor marker kit results were all within normal limits; TORCH nucleic acid test result was negative. The patient on the admission was diagnosed with HSP nephritis.

**Table 1:** Laboratory test results.

Parameter	Test results							Normal range
	D 1	D 5	D 6	D 7	D 12	D 27	D 28	
WBC (×10^9^/L)	16.7	13.9	8.3	7.9	4.8	9.8	5.1	3.6–9.7
NEUT (%)	90	65	69	78	65	62	66	23.6–75
PLT (×10^9^/L)	236	19	24	12	75	65	130	100–450
Hb (g/L)	134	74	69	80	58	100	92	110–146
CRP (mg/L)	1.4	2.7	9.2	4.2	20.7	3.6	2.8	0–8
PCT (ng/mL)	0.17	3.72	NA	5.71	0.58	NA	NA	<0.05
PT (s)	14.4	18.1	15.5	13.1	11	13.7	13.0	7.6–13.6
APTT (s)	26.1	66.9	44.8	34.3	35	27.3	27.3	16.9–36.9
Fg (mg/dL)	184	147	143	93	225	88	70	200–400
FDP (μg/mL)	16.5	82.9	91.8	81.9	94	NA	NA	< 5
DDI (mg/l)	7.28	46.09	38.19	29.52	60.08	NA	NA	< 0.55

APTT = activated partial thromboplastin time, CRP = C-reactive protein, DDI = d-dimers, FDP = fibrin degradation products, Fg = fibrinogen, Hb = hemoglobin, NA = not available, NEUT = *neutrophils*, PCT = procalcitonin, PLT = platelets, PT = prothrombin time, WBC = white blood cells.

**Figure 1.**
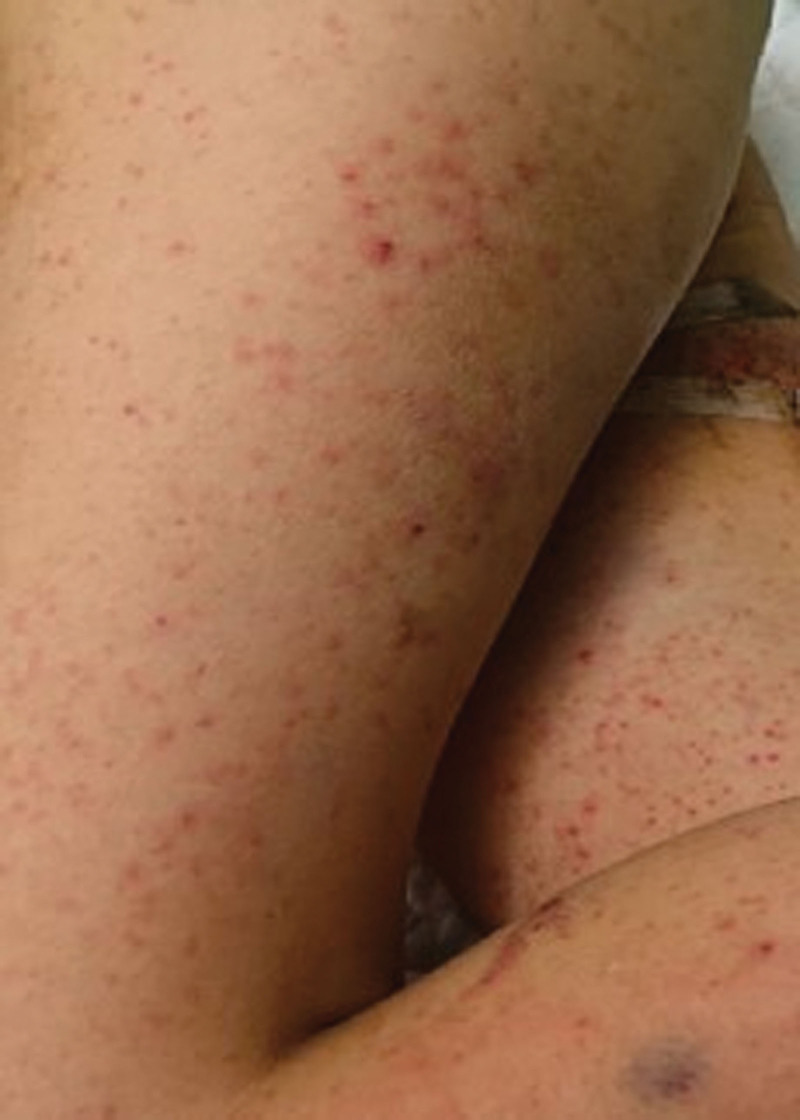
Nascent skin rash on lower limbs in the patient on the 3rd hospital day.

On the 1st hospital day, she was started on methylprednisolone (1 mg/kg, bid) for anti-inflammation, dipyridamole for anti-platelet (PLT) aggregation, cimetidine for stomach protection, and calcium supplementation. On the 2nd day, the child condition had no improvement. Supplementary tests for amylase and lipase as well as chest and abdomen CT showed no abnormality. On the 3rd day, because of the severity of the child recurrent abdominal pain accompanied by nascent skin rash (Fig. 1) and eyelid edema, she was given hemoperfusion at noon. She received methylprednisolone pulse therapy in the afternoon (10 mg/kg). After the therapy, the child still had persistent abdominal pain with progressed skin rash. On the 4th day, the patient received the first plasma exchange on the morning. But in the afternoon, the child began to have fever up to 39.4°C. Blood routine examination showed a white blood cell count 13.9 × 10^9^/L (3.6–9.7 × 10^9^/L) (reference range is in parentheses hereafter), neutrophils 70% (23.6%–75%), hemoglobin 106 g/L (110–146 g/L), PLT count 31 × 10^9^/L (100–450 × 10^9^/L), C-reactive protein 2.6 mg/L (0–8 mg/L), procalcitonin 0.52 ng/mL (<0.05 ng/mL). The blood coagulation function was not examined. Because of the concern for severe infection, the patient was treated with meropenem for anti-bacterial infection and gamma-globulin (20 g) for immuno-support.

**Figure 2.**
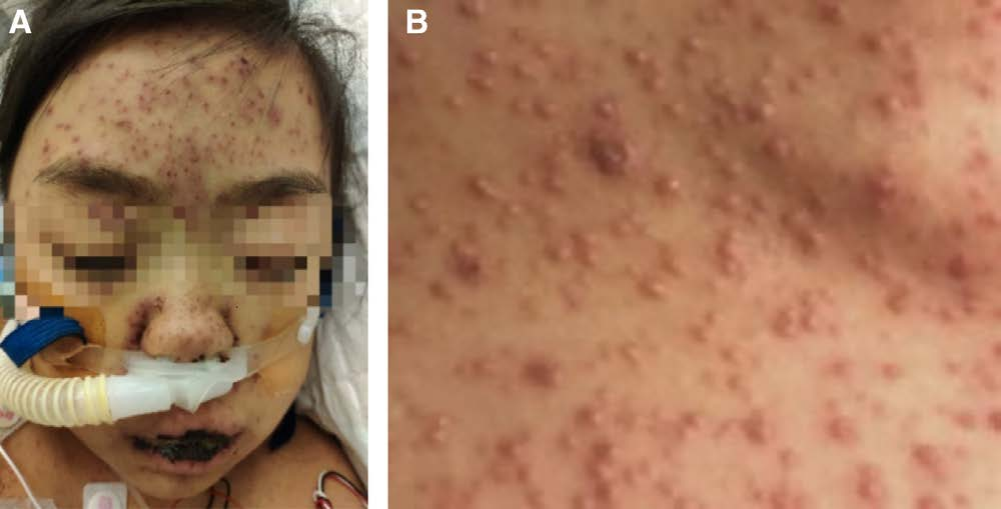
Vesicular rash on the skin of the face and the back on the 5th hospital day. (A) Facial vesicular rash, ulceration, crusted lesions, and erosion, as well as bleeding on her lips; (B) vesicular rash on the skin of the back.

On the 5th day, the patient had persistent fever accompanied by strong abdominal and lower back pain, epistaxis, erosion and bleeding on her lips and in her mouth. In addition, she developed purpuric maculopapules, partial ulceration, crusted lesions, and vesicular lesion eruption on the surface of the extensive purpuric rash on her face (Fig. 2A), auricle, chest, back (Fig. 2B) and limbs. She also had hematochezia. Slight bleeding was noted at the sites where the hemodialysis catheter and indwelling needle were placed. Blood routine examination showed multiple abnormalities including increased white blood cell 13.9 × 10^9^/L, further decreased hemoglobin 74 g/L, dramatically decreased PLT 19 × 10^9^/L, and dramatically increased procalcitonin 3.72 ng/mL. The coagulation function test also revealed obvious abnormalities including increased prothrombin time (PT) 18.1 second (7.6–13.6 seconds), activated partial thromboplastin time (APTT) 66.9 seconds (16.9–36.9 seconds), fibrin degradation products 82.9 μg/mL (<5 μg/mL), d-dimers 46.09 mg/L (<0.55 mg/L), and decreased fibrinogen (Fg) 147 mg/dL (200–400 mg/dL). Moreover, laboratory results showed elevated amylase 187 U/L (30–110 U/L) and lipase 1007 U/L (23–300 U/L). Abdominal CT confirmed pancreatitis, peritonitis, and ascites. Liver function examination showed significantly increased hepatic enzymes alanine aminotransferase 1967 U/L (9–52 U/L) and serum aspartate aminotransferase 2785 U/L (14–36 U/L). Therefore, the patient was additionally diagnosed with sepsis, DIC with acute liver failure, pancreatitis, and possible varicella. Plasma sample was collected for detecting VZV nucleic acids. She was given metopropenem for anti-bacterial infection; acyclovir for anti-VZV; methylprednisolone for anti-inflammation; infusion with fresh frozen plasma, cryoprecipitation, prothrombin complex, irradiated PLTs, and vitamin K1 for improving blood coagulation function; polyene phosphatidylcholine for liver protection; somatostatin and omeprazole for gastric acid suppression and stomach protection; albumin, fasting, fluid replacement, and other supportive treatment for symptom management. On the evening of the 5th day, the child developed shortness of breath (with a respiratory rate of 38/minute) and a deteriorated systemic bleeding tendency accompanied by drowsiness. She was then transferred to intensive care unit (ICU) for further treatment.

On the 6th day, the patient was started on continuous renal replacement therapy (CRRT) and plasma exchange for 3 days in ICU. On the 7th day, the patient VZV nucleic acid result turned out to be positive. After consultation with Department of Infection of our hospital, the patient was started on intravenous acyclovir (10 mg/kg/8 hours) for 3 weeks, and methylprednisolone was gradually discontinued in a week. After consultation with Department of Pharmacy, the use of antibiotics during ICU stay were adjusted multiple times from meropenem to cefoperazone sulbactam, levofloxacin, linezolid, tigecycline, fluconazole, and micafungin. On the 9th day, the patient was started on intravenous gamma-immunoglobulin (400 mg/kg/day) for 5 days.

During ICU stay, the patients received multiple times of transfusion of red blood cells, PLTs, fresh frozen plasma, cryoprecipitate, prothrombin complex, and Fg (Supplementary Table 1, http://links.lww.com/MD/K782) to treat systemic bleeding and improve blood coagulation function. The PLTs of the child began to increase to ≥ 50 × 10^9^/L on the 12th hospital day from a nadir of 12 × 10^9^/L on the 7th day. PT returned to normal range on most of the tests after the 9th day; APTT began to be stabilize within normal range from the 12th day. The symptoms of bleeding gradually improved concomitantly.

On day 25, the patient was transferred from ICU back to Nephrology Department. The use of antibiotics and acyclovir was gradually discontinued in a week. No fever or bleeding was observed since then. Due to the urination analysis results showing hematuria and proteinuria, the patient was started on methylprednisolone to treat inflammation on day 29. Based on the improvement in her condition, she was discharged on day 37.

One year after the discharge, the child blood routine tests, urination analyses, and renal function tests were all normal, and medications such as oral methylprednisolone were discontinued. The child was followed up without recurrence or any complication 2 years after discharge.

## 3. Discussion

To the best of our knowledge, this is the first report on varicella-associated DIC secondary to HSP with renal and GIS involvement in a child. Although it is known that immunosuppressed patients with renal diseases are at an increased risk for complications due to varicella, the severity of the varicella-associated complications reaching the level of DIC secondary to HSP in a child has never been reported, and the patient was successfully rescued with prompt aggressive multiple-modality therapy without any complication 2 years after discharge. DIC is a life-threatening complication, which results from an overwhelming activation of coagulation leading to: microvascular fibrin thrombi which can cause multi-organ dysfunction syndrome from tissue ischemia, and consumption of PLTs, clotting factors, Fg, and thereby life-threatening hemorrhage.^[3–5]^ Laboratory abnormalities in DIC include decreased PLT count and Fg, prolonged APTT and PT, as well as increased fibrin degradation products including d-dimers (plasmin degradation of cross-linked fibrin). DIC has a high mortality rate of 45% to 78%.^[3]^

DIC can be provoked by multiple underlying clinical conditions such as sepsis or severe infection, trauma, organ destruction, obstetrical calamities, cancer, severe toxic or immunological reactions.^[4,5]^ VZV infection can lead to severe complications including DIC mostly in immunocompromised patients or in individuals with protein C and protein S deficiency. However, there are some critical differences in the clinical manifestations of varicella-associated DIC in immunocompromised patients^[6–17]^ and in individuals with protein C and protein S deficiency which causes purpura fulminans.^[18–27]^ Purpura fulminans is a highly thrombotic subtype of DIC characterized by the overt presentation of erythematous macular skin lesions on the trunk and extremities.^[18–28]^

As previously reported, the immunocompromised patients who developed varicella-associated DIC included recipients of bone marrow, hematopoietic stem cell, or organ transplantation, patients with primary nephropathy who were receiving corticosteroid therapy, cancer patients receiving chemotherapy, and patients with human immune deficiency virus infection.^[6–17]^ The case reported here is novel because, to our knowledge, there has been no report of VZV-associated DIC developed 24 days after the onset of HSP that has no usual initial clue of varicella.

HSP, a common form of small vessel vasculitis in children, is characterized by nonthrombocytopenic palpable purpura, arthritis, renal and GIS involvement. Although HSP is usually triggered by an antigenic stimulus including infectious agents, drugs, vaccines, cold, an insect bite or foods, it is rarely triggered by VZV infection in children.^[29]^ In previous reports on varicella-associated HSP, varicella occurred prior to or concomitantly with the onset of HSP.^[30–34]^

Our patient on admission did not have typical presentations of varicella and DIC. She was admitted with severe HSP with renal and GIS involvement, but with a normal body temperature, a normal PLT count, and no signs of bleeding. Since the use of corticosteroids is the mainstay in the treatment of severe HSP, she received treatments including methylprednisolone before and shortly after the admission to our hospital. However, the treatment did not have obvious effect in improving the condition of the patient. Based on the severity of the patient disease and the experts consensus on “Evidence-based Diagnosis and Treatment Recommendations for Children with Henoch-Schönlein Purpura” in China, the patient was given hemoperfusion and plasma exchange once each. The treatments were not enough for stopping the progression of the disease. We judged that the methylprednisolone treatment might have caused immunosuppression, hence facilitated the reactivation or acquisition of severe VZV infection that caused DIC. It was found that the risk of severe varicella in patients receiving steroid treatment was 178 times higher than that in the general population.^[35]^ In our patient, since VZV was not analyzed before the appearance of typical vesicular skin rash, we could not absolutely exclude another possible that the initial HSP with gastrointestinal involvement was already an unusual manifestation of undetected VZV infection,^[36]^ which progressed to more severe VZV infection a few days after admission to our hospital. Yet this possibility might be low because the appearance of varicella-indicating vesicular skin rash would lag behind the initial unusual gastrointestinal manifestation for only about 24 to 96 hours.^[37]^ The possibility that the patient had tumors and other diseases leading to the abnormal blood coagulation, such as systemic lupus erythematosus, anti-neutrophil cytoplasmic antibodies-associated vasculitis, primary thrombocytopenia, protein C and protein S deficiency, were excluded based on our laboratory results.

Given the high mortality rate in patients with DIC, early diagnosis of underlying trigger and prompt aggressive treatment are critical to rescuing those patients. A previous review of immunocompromised patients showed that the mean time from initial symptoms of varicella-associated multi-organ dysfunction to start of treatment was 4.2 days for the patients who survived, whereas a mean time of 5.5 days was reported for those who died, demonstrating the importance of early diagnosis and prompt treatment.^[38]^ Even in some cases with early diagnosis, delayed aggressive treatment had fatal outcomes, some times within a few days.^[3,7]^ For our patient, acyclovir was administered on the same day as the appearance of vesicular skin rash and the diagnosis of DIC with suspected varicella, without waiting for the outcome of the VZV nucleic acid analysis (which turned out to be positive 2 days later). Acyclovir can stop the replication of the VZV by competitively inhibiting the viral DNA polymerase, and is usually the first choice for the treatment of varicella. In addition, fresh frozen plasma, cryoprecipitation, prothrombin complex, irradiated PLTs, and vitamin K1 was given for improving blood coagulation function; medications for liver and GIS protection were also administered on the same day. Even with the above treatment, the patient condition worsened on that evening and she was transferred to ICU. On the next day, CRRT and plasma exchange was started, and continued for 3 days. The treatments attempted to remove toxins and bring the patient plasma back to its homeostatic milieu. It has been also reported that plasma exchange could precipitously reduce serum VZV concentrations.^[8]^ Meanwhile, after the confirmation of VZV infection by nucleic acid analysis, methylprednisolone was gradually discontinued in a week (the tapering was to avoid the adrenal crisis caused by sudden stop of the hormone supply). Moreover, it is known that gamma-immunoglobulin can reduce the inflammatory reaction, block the Fc receptor of monocyte-macrophage, prevent the activation of T cells, neutralize endogenous antiplatelet antibody, and has the functions of virus neutralization and inhibition of virus proliferation. Therefore, intravenous gamma-immunoglobulin treatment was started a day before the diagnosis of varicella-associated DIC and maintained for 5 days following CRRT plus plasma exchange after the DIC diagnosis. This was also believed to be crucial for shortening the course of disease and preventing fatal complications. Other supportive treatments for the patient in ICU included multiple times of transfusion of red blood cells, PLTs, fresh frozen plasma, cryoprecipitate, prothrombin complex, and Fg to improve blood coagulation function. Antibiotics was also administered because it is known that bacterial superinfection is one of the most common severe complications of varicella. After the multi-modality treatment, the child finally recovered. She has been followed up for about 2 years without any relapse.

Our study existed some limitations. Firstly, skin biopsy of the patient on admission was not performed to detect if there was VZV infection in the first place. Secondly, kidney biopsy was not performed at our hospital to diagnose HSP nephritis and to further confirm whether there was a microvascular thrombus in the kidney. Thirdly, we did not further examine PLT function and the level of related factors such as protein S, protein C and other clotting factors.

In conclusion, this report serves as a novel reminder that patients with HSP receiving corticosteroid treatment may have a risk of suffering from life-threatening secondary varicella-associated DIC with severe thrombocytopenia and multi-organ dysfunction syndrome. Early identification of varicella and prompt intensive treatment with antiviral, gamma-immunoglobulin, plasma exchange, and other integrative treatments for improving blood coagulation function together with gradual discontinuation of the steroid use are essential life-saving approaches.

## Author contributions

**Conceptualization:** Hui Guo.

**Data curation:** Kai Liao, Xiu-Ying Chen.

**Supervision:** Hui Guo.

**Writing – original draft:** Jing Jiang.

**Writing – review & editing:** Jing Jiang.

## Supplementary Material


